# Temporal Stability and Genetic Diversity of 48-Year-Old T-Series Phages

**DOI:** 10.1128/mSystems.00990-20

**Published:** 2021-02-16

**Authors:** Dinesh Subedi, Jeremy J. Barr

**Affiliations:** a School of Biological Sciences, Monash University, Clayton, Victoria, Australia; CSIRO

**Keywords:** bacteriophages, T series, bacteriophage T4, stability, genetic drift, T4, bacteriophage evolution, bacteriophage genetics, bacteriophages

## Abstract

T-series phages have been model organisms for molecular biology since the 1940s. Given that these phages have been stocked, distributed, and propagated for decades across the globe, there exists the potential for genetic drift to accumulate between stocks over time. Here, we compared the temporal stability and genetic relatedness of laboratory-maintained phage stocks with a T-series collection from 1972. Only the T-even phages produced viable virions. We obtained complete genomes of these T-even phages, along with two contemporary T4 stocks. Performing comparative genomics, we found 12 and 16 nucleotide variations, respectively, in the genomes of T2 and T6, whereas there were ∼172 nucleotide variations between T4 sublines compared with the NCBI RefSeq genome. To account for the possibility of artifacts in NCBI RefSeq, we used the 1972 T4 stock as a reference and compared genetic and phenotypic variations between T4 sublines. Genomic analysis predicted nucleotide variations in genes associated with DNA metabolism and structural proteins. We did not, however, observe any differences in growth characteristics or host range between the T4 sublines. Our study highlights the potential for genetic drift between individually maintained T-series phage stocks, yet after 48 years, this has not resulted in phenotypic alterations in these important model organisms.

**IMPORTANCE** T-series bacteriophages have been used throughout the world for various molecular biology researches, which were critical for establishing the fundamentals of molecular biology, from the structure of DNA to advanced gene-editing tools. These model bacteriophages help keep research data consistent and comparable between laboratories. However, we observed genetic variability when we compared contemporary sublines of T4 phages to a 48-year-old stock of T4. This may have effects on the comparability of results obtained using T4 phage. Here, we highlight the genomic differences between T4 sublines and examined phenotypic differences in phage replication parameters. We observed limited genomic changes but no phenotypic variations between T4 sublines. Our research highlights the possibility of genetic drift in model bacteriophages.

## INTRODUCTION

Bacteriophages, viruses that infect bacteria, were discovered in the early 20th century. The antimicrobial properties of phages initially sparked the interest of early phage pioneers, and phages were quickly used to treat bacterial infections, such as dysentery and cholera (1). However, the ambiguous efficacy of phage therapy, along with the introduction of more efficient chemotherapeutics, led to the subsequent decline in interest in their use as therapeutic agents (2). Nevertheless, since their discovery, phages have become key model organisms to understand various aspects of modern molecular biology. For example, the understandings of the basis of mutation (3), recombination (4), the genetic nature of DNA and its replication (5), and the sequencing of genes and genomes (6) were all founded upon phage biology. Furthermore, the study of phage prokaryote resistance mechanisms led to the discovery of the CRISPR (clustered regularly interspaced palindromic repeats)/Cas system, which has become a key technique for targeted mutagenesis and gene editing (7, 8). Nowadays, amid the looming threat of antibiotic resistance, phage therapy has regained global attention (9).

One of the most important historical advancements in phage biology was the introduction of the T-series phages in the 1940s by Delbruck and colleagues—the so-called “phage group.” This enabled phage researchers to compare results between different laboratories, which was previously unsystematic, due to the data coming from a random collection of phage-host combinations. T-series phages constitute a collection of seven virulent phages (type 1 [T1] to T7), which were described based on their ability to lyse Escherichia coli B (3, 10). Although this classification was based merely on the lytic activity of the group, T2, T4, and T6 (the T-even series) turned out to be similar morphologically, antigenically, and genetically (11). T-even phages are classified as members of the family *Myoviridae* with their characteristic contractile tail. The genomes of T-even phages contain between 160 and 170 kbp double-strand DNA (dsDNA), which has 5-hydroxymethyl-cytosine in place of cytosine (12). In contrast, T-odd phages (T1, T3, T5, and T7) are highly variable relative to each other. They have a relatively simple noncontractile tail and, unlike the T-even series, contain dsDNA with the usual four nucleotides. Genome size is also diverse among the T-odd phages (T1, 48 kbp; T3, 38 kbp; T5, 121 kbp; T7, 40 kbp). The traditional T-series phages are still in use worldwide. They have been distributed, maintained, and propagated across many different laboratories and repositories for decades. However, questions remain regarding the stability and genetic drift of model T-series bacteriophages over time and how these changes have shaped bacterial hosts, past and present.

**FIG 1:**
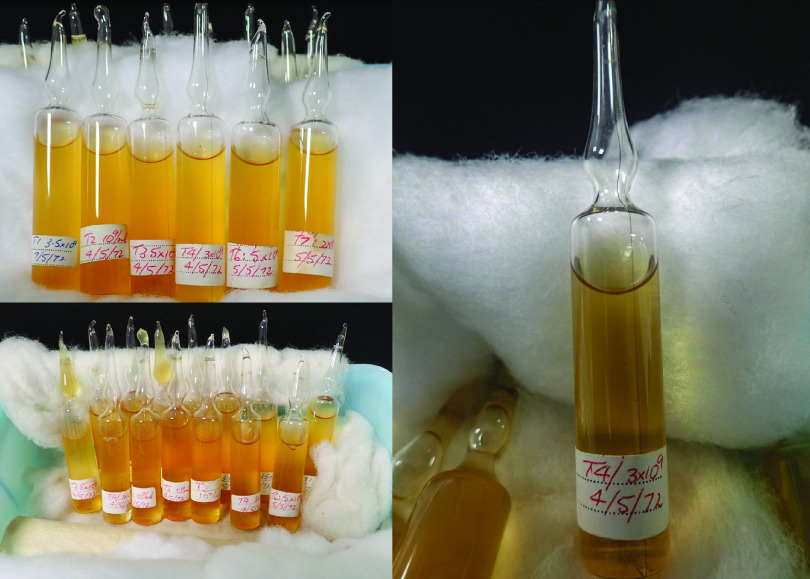
Photographs of historical stocks of T-series phages, stored in 1972. Each glass ampule contained 10 ml lysate stored in chloroform-sealed glass ampules at a titer of 1 × 10^9^ to 5 × 10^9^ PFU/ml. The lysates have been stored at 4°C since 1972.

We therefore sought to examine the temporal stability of T-series phages in prolonged storage and compare the genetic relatedness of different laboratory-maintained T4 phage stocks. We obtained a nearly complete T-series phage stock (T1, T2, T3, T4, T6, and T7; notably, T5 was missing) that had been prepared by Robert E. Hancock and coworkers at the University of Adelaide, Australia, in 1972 (13, 14). The T-series lysates had been purified and stored in chloroform-sealed glass ampules (Fig. 1). To evaluate the stability of these phages, we quantified active phages in the ampules by standard top-agar assays. To compare the genetic divergence of the phages, we obtained two contemporary stocks of T4 (referred to here as T4 sublines for simplicity) from phage laboratories in Australia and the United States. We then compared the genetic and phenotypic differences of these sublines to Hancock’s 1972 stock of T4.

## RESULTS

### Stability of T-series phages in prolonged storage.

To examine the viability of the 48-year-old T-series phage stocks, we plated 1 ml of lysate from T2, T3, T4, T6, and T7 phages with their respective hosts using the soft-agar overlay technique. We did not open or plate T1 phage vials due to concerns about its persistence and history of contaminating laboratory stocks of E. coli. The titers, in PFU per milliliter, were recorded from at least three different vials for each strain (Table 1). We did not observe any plaques from T3 and T7 lysates. To verify whether there were any active phage particles in the T3 or T7 stocks remaining at low titers, we propagated entire vials of the original lysates with E. coli B and E. coli BL21, respectively, overnight in an attempt to recover viable phages. However, no phages could be recovered following overnight amplification. Lysates of T2 and T4 showed 4 to 6 plaques per ml on top agar, while T6 phage had 10^4^ to 10^5^ PFU/ml (Table 1). Our results revealed that T-even series phages could be revived from the lysates stored approximately 48 years ago, while T-odd series phages (T3 and T7) were not able to be recovered from the 1970s stock.

**TABLE 1:** Growth of T-series phages from 48-year-old stocks

Bacteriophage	Growth (PFU/ml) in:			Mean ± SD (PFU/ml)
	Ampule 1	Ampule 2	Ampule 3	
T2	4	3	3	3.34 ± 0.47
T3	No growth	No growth	No growth	
T4	3	6	2	3.67 ± 1.7
T6	4.2 × 10^4^	3.1 × 10^5^	6.2 × 10^4^	1.38 × 10^5^ ± 1.21 × 10^5^
T7	No growth	No growth	No growth	

### Genomic characteristics of the T-even series.

The complete genomes of 1972 stocks of T2, T4, and T6 phage, referred to here as Hancock phages, were obtained using Illumina HiSeq. Raw reads of each genome were aligned with the corresponding NCBI RefSeq sequences to examine single nucleotide polymorphisms (SNPs) and insertion-deletion variations (indels) using Snippy v4.2.0. We observed 12 and 16 variations (SNPs plus indels) in the T2-Hancock and T6-Hancock phages, respectively, compared with the NCBI RefSeq sequences (T2 nucleotide accession no. LC348380; T6 nucleotide accession no. MH550421) with the nucleotide variations being distributed throughout the genome (Data Set S1, sheets 4 and 5). Comparatively, when we investigated the T4-Hancock phages, we found 172 nucleotide variations compared to the NCBI T4 RefSeq sequence (nucleotide accession no. AF158101). The reason for this high degree of nucleotide variation was not immediately clear. The original T4 genome sequence in the NCBI database was last updated in 2003 and had been completed using traditional sequencing methods involving cloning and PCR (15). To examine whether this difference arose due to artifacts in T4’s RefSeq entry, we compared genome sequences of two contemporary T4 sublines from the Barr lab (Monash University, School of Biological Sciences, Australia) (referred to here as T4-Barr) and from the Bacteriophage T4 lab, led by Venigalla B. Rao, The Catholic University of America, Washington, DCs, USA (referred to here as T4-Rao) to the NCBI RefSeq entries. We observed that T4-Barr and T4-Rao had similar numbers of nucleotide variations, 172 and 175, respectively, compared with the T4 RefSeq entry (Data Set S1, sheets 1, 2, and 3). This led us to conclude that there may be artifacts in the NCBI RefSeq genome, and we therefore used T4-Hancock as a historical reference genome in our subsequent analysis.

10.1128/mSystems.00990-20.2 DATA SET S1 Sheets 1, 2, and 3 show nucleotide variations in T4-Barr, T4-Hancock, and T4-Rao, respectively, compared to NCBI GenBank reference genome *Escherichia* phage T4 (AF158101). Sheets 4 and 5 show nucleotide variations in T2-Hancock and T6-Hancock, respectively, compared to NCBI GenBank reference genome *Escherichia* phage T2 (LC348380). Sheet 6 shows nucleotide variations of T4-like genomes in the NCBI database compared to T4-Hancock (+, presence of variation; orange indicates variations shared with T4-Barr and T4-Rao). Download Data Set S1, XLSX file, 0.1 MB. Copyright © 2021 Subedi and Barr. 2021 Subedi and Barr. https://creativecommons.org/licenses/by/4.0/ This content is distributed under the terms of the Creative Commons Attribution 4.0 International license.

### Comparative genomics of T4 sublines.

**FIG 2:**
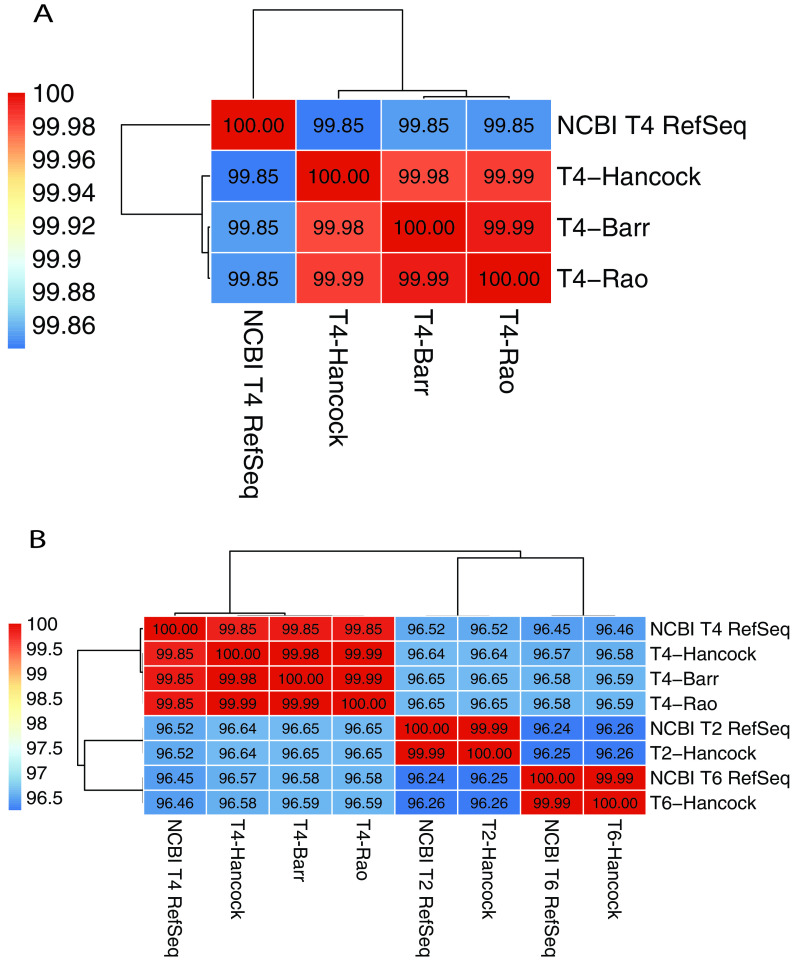
Average nucleotide identity (ANI) matrix of T-series phages. (A) ANI matrix of T4 sublines (NCBI T4 RefSeq refers to the GenBank accession number AF158101). (B) ANI matrix of T-even phages (GenBank accession numbers for NCBI RefSeq entries: T4, AF158101; T2, LC348380; T6, MH550421). Dendrograms represent sequence similarity between genomes. The bars on the left show the color codes for percentage similarity.

To compare the complete genomes of T-even phages, we assembled the Illumina HiSeq reads using Unicycler v0.4.3 with a minimum contig length of 1,000 bp, which produced a single contig of a complete genome for each strain. The genome size of T-even series of Hancock strains was broadly similar to that of the respective NCBI RefSeq entry (Table 2). The complete genomes were aligned by pairwise sequence alignment as implemented in Pyani (https://huttonics.github.io/pyani/) to obtain the average nucleotide identity (ANI). Analysis revealed that genomes of T-even phages were closely related to each other, with more than 96% nucleotide sequence similarity between T2, T4, and T6 phages (Fig. 2B). T4-Barr and T4-Rao showed higher similarity to each other than to T4-Hancock (Fig. 2A).

**TABLE 2:** Genome size and nucleotide variations of T-series phages compared with NCBI RefSeq genomes

Strains	Genome size (bp)	NCBI RefSeq accession no. (genome size [bp])	No. of nucleotide variations (SNPs + indels)
T2-Hancock	163,826	LC348380 (163,825)	12 (8 + 4)
T4-Hancock	168,908	AF158101 (168,903)	172 (97 + 75)
T4-Barr	168,921	AF158101 (168,903)	172 (99 + 73)
T4-Rao	168,922	AF158101 (168,903)	175 (100 + 75)
T6-Hancock	168,702	MH550421 (168,706)	16 (8 + 8)

Next, we assessed the nucleotide variations (SNPs and indels) between T4 sublines. To obtain an unbiased annotation, we manually annotated the complete genome of each subline using information from NCBI T4 RefSeq with an identity threshold of at least 98%. T4-Barr had two insertions (frameshift) and six SNPs (five missense and one synonymous) compared to T4-Hancock. Our analysis showed five additional variations (one insertion and four missense SNPs) in T4-Rao, taking the total number of variations to 13 (Table 3).

**TABLE 3:** Nucleotide variations in T4-Barr and T4-Rao compared to T4-Hancock as the reference^*a*^

Gene	Product	Variation		Type of variation	Function category^*b*^
		Nucleotide	Amino acid		
rIIA	Protector from prophage-induced early lysis	222A→C	Glu74Asp	Missense	Auxiliary
39	Topoisomerase II, large subunit, N-terminal region	475A→G	Ile159Val	Missense	Essential
dda	DNA helicase	1174G→A	Ala392Thr	Missense	Auxiliary
55.2	Hypothetical protein	123A→G	Gln41Gln	Synonymous	
rI.-1	**MobD.6 hypothetical protein**	**179dupG**	**Glu61fs**	**Frameshift**	
tk.4	Conserved hypothetical protein	106A→G	Ser36Gly	Missense	
7	Baseplate wedge initiator	776T→C	Phe259Ser	Missense	Essential
—^*c*^	Intragenic region				
18	**Tail sheath protein**	**326G→A**	**Ser109Asn**	**Missense**	**Essential**
alt	**Alt RNA polymerase ADP-ribosylase**	**691C→T**	**Arg231Cys**	**Missense**	Auxiliary
34	Long tail fiber, proximal subunit	1151C→T	Pro384Leu	Missense	Essential
37	**Long tail fiber, distal subunit**	**1250G→A**	**Gly417Asp**	**Missense**	**Essential**
arn	Arn inhibitor of MrcBC restriction endonuclease (anti-restriction nuclease)	258dupA	Leu87fs	Frameshift	Auxiliary
52	**Topoisomerase II, medium subunit**	**757G→A**	**Asp253Asn**	**Missense**	**Essential**

a Bold typeface denotes variations present in T4-Rao but absent in T4-Barr.

b Insertion of GAAGCTTTGGCCC at the intergenic region at 93,397 bp.

c Assigned according to reference 15.

Frameshift mutations were observed in an auxiliary gene (*arn*) and a hypothetical protein (MobD.6). *arn* encodes an anti-restriction endonuclease that inhibits the E. coli host’s endonuclease activity (16). Our manual annotation of T4-Hancock showed that Arn consists of 94 amino acids, which is slightly different from that of NCBI T4 RefSeq (92 amino acids). Interestingly, duplication of nucleotide A at position 258 of *arn* resulted in a stop codon at position 276, truncating the protein length to 92 amino acids in T4-Barr and T4-Rao compared to 94 amino acids in T4-Hancock. The frameshift mutation in MobD.6 also caused a gain of a premature stop codon at position 180 (of 387 bp), potentially resulting in a loss-of-function mutation in this hypothetical gene.

On the other hand, many missense variations were observed in genes associated with essential structures, such as two units of topoisomerase II (gp39 and gp52), which helps in DNA metabolism, and the baseplate wedge initiator (gp7), tail sheath protein (gp18), and long tail fibers (gp34 and gp37), all associated with phage adsorption to the bacterial host. Of the five additional variations in T4-Rao, three were observed in essential genes: the tail sheath protein (gp18), distal subunit of long tail fiber (gp37), and medium subunit of topoisomerase (gp52).

We also investigated variations in the genome of T4 or T4-like phages available in NCBI databases compared to that of T4-Hancock. At first, we interrogated the NCBI nucleotide database using the keywords “Escherichia phage T4” or “Escherichia virus T4” and “complete genome.” We received eight entries with complete genomes (including T4-Hancock of our study and T4-RefSeq) from our search criteria as of 15 December 2020. Furthermore, we included other T4-like genomes from published papers (17–20), making a total of 10 complete genomes for the comparison. On nucleotide comparison, we obtained identities between 82.9% and 99.9% among 10 genomes (Fig. S1). Next, we selected six genomes that showed >99% similarity for variant analysis. We observed a total of 89 nucleotide variations in various coding sequences (CDS) among those six genomes compared to T4-Hancock (Data Set S1, sheet 6) and this also includes 12 variations (of 13) observed in the genomes of T4-Barr and T4-Rao.

10.1128/mSystems.00990-20.1 FIG S1 Average nucleotide identity of 10 T4 and T4-like phage genomes. Dendrograms represent sequence similarity between genomes. The bar on the right shows the color code for percentage similarity. GenBank nucleotide accession numbers are shown at the bottom. Download FIG S1, PDF file, 0.4 MB. Copyright © 2021 Subedi and Barr. 2021 Subedi and Barr. https://creativecommons.org/licenses/by/4.0/ This content is distributed under the terms of the Creative Commons Attribution 4.0 International license.

### Growth characteristics of T4 sublines in E. coli B and E. coli K-12.

**FIG 3:**
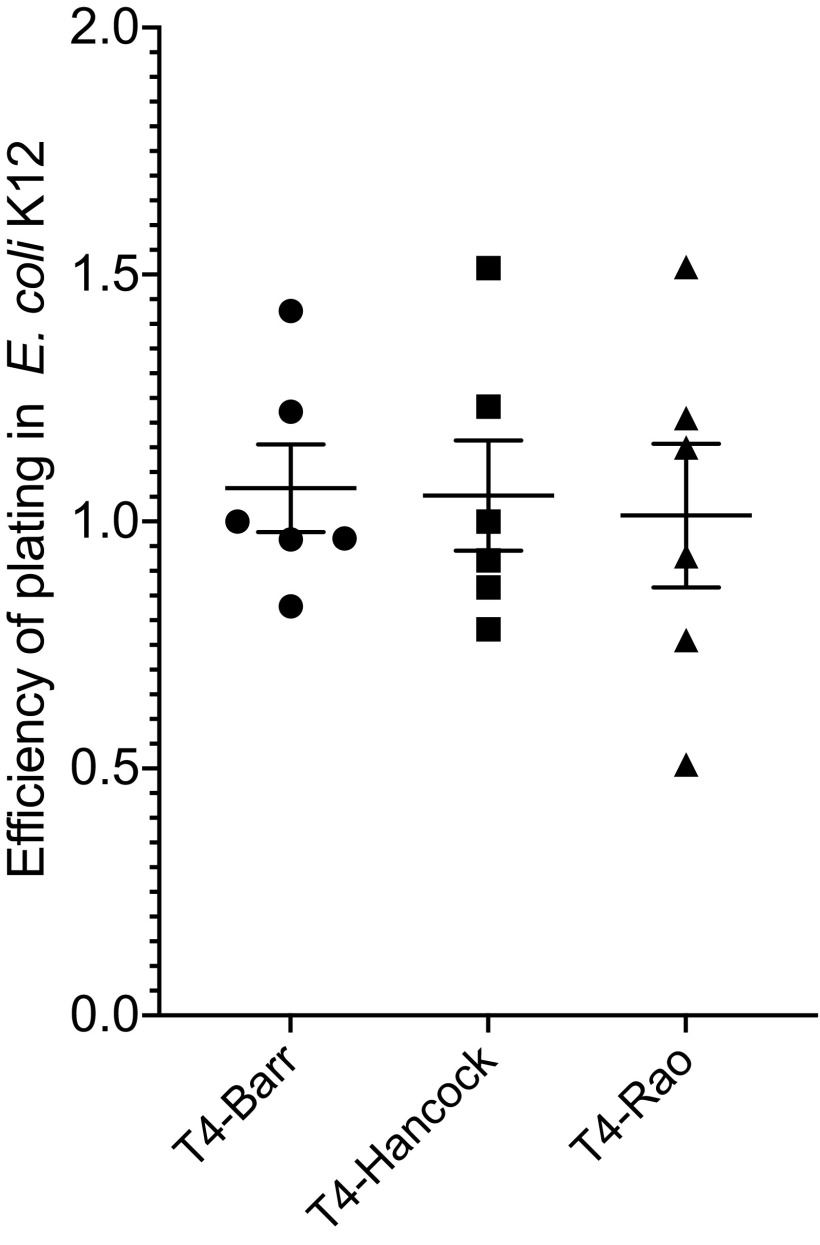
Efficiency of plating (EOP) for T4 sublines on E. coli K-12. Each experiment was performed in duplicate and repeated three times on separate occasions. The values in the graph are means for six replicates, and error bars represent standard errors of the means (*P* = 0.91).

We then sought to assess whether the identified mutations had effects on phage replication variables. For these experiments, we used two hosts: E. coli B and the closely related strain E. coli K-12. T4 adsorbs to these two E. coli strains using different receptors. In E. coli K-12, T4 uses lipopolysaccharide (LPS) and outer membrane protein C (OmpC) as receptors. However, in *E coli* B, which harbors a deletion in *ompC*, T4 uses LPS as the sole receptor (21, 22). First, we performed an efficiency-of-plating (EOP) assay to examine relative titers of T4 sublines on E. coli K-12 compared to the original host of propagation, E. coli B. Our results showed that the relative EOP were not significantly different between any of the three sublines of T4. However, T4-Rao had slightly lower EOP (1.0) than T4-Hancock (1.1) and T4-Barr (1.1) (Fig. 3).

**FIG 4:**
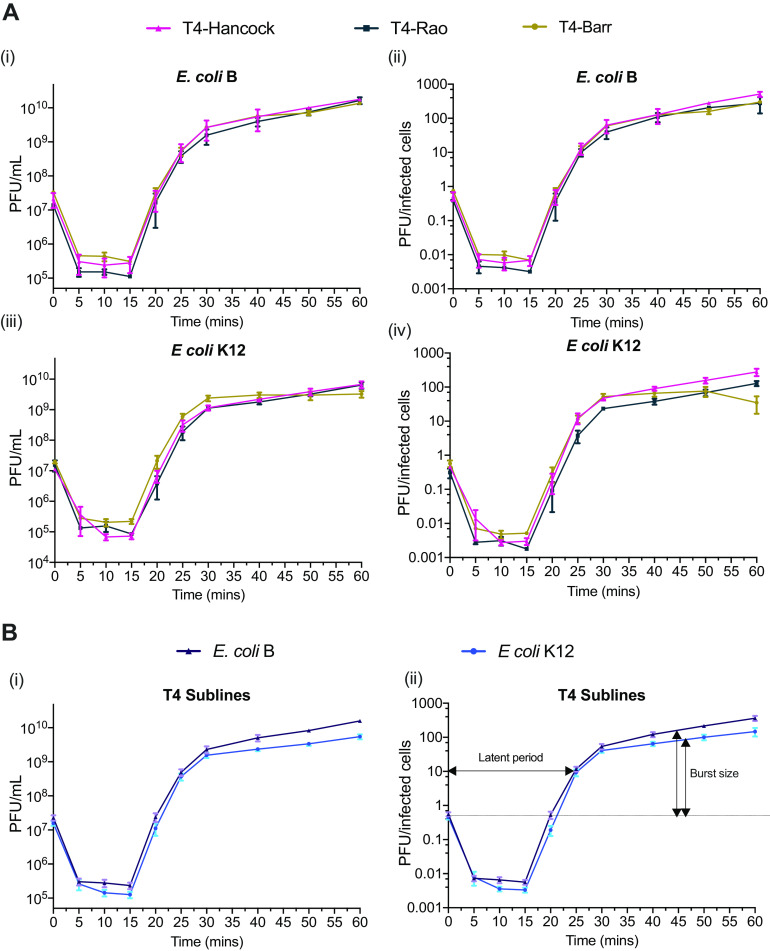
One-step growth curve of T4 sublines (A) Growth curves of T4 sublines in E. coli B (i and ii) and E. coli K-12 (iii and iv). The values in panels ii and iv were calculated by dividing the number of PFU at each time point by CFU of the initial inoculum. (B) Growth curve representing the averages for all three T4 sublines in E. coli B (i) and E. coli K-12 (ii). All experiments were repeated in triplicate on three separate occasions. Error bars represent standard errors of the mean.

Next, we compared the replication cycle variables of latency period and burst size using one-step growth curves on the same pair of hosts. Our results revealed that all T4 sublines had similar growth features, characterized by a latent period of approximately 20 min, followed by a maturation period of ∼10 min, reaching their plateau within ∼30 min (Fig. 4) as described previously (23). Similarly, the average burst sizes of all the T4 sublines were comparable within the two bacterial hosts. However, the burst size in E. coli B was calculated to be approximately 190 phage particles per infected bacterial cell, which was significantly higher than the average burst size of 109 phage particles per infected bacterial cell in E. coli K-12.

**FIG 5:**
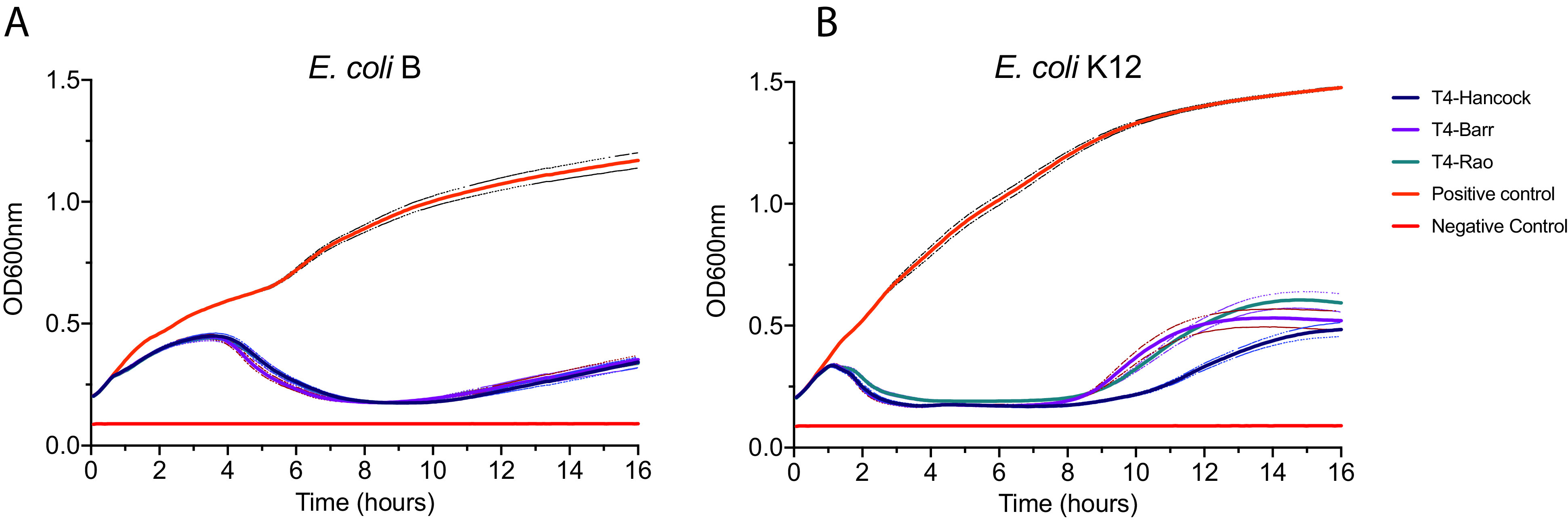
Growth kill curve of T4 sublines on E. coli B (A) and E. coli K-12 (B) showing lytic activity of T4 phages for 16 h. The OD_600_ was collected at 5-min intervals, and each point in the graph is the mean for 36 replicates (12 technical replicates in three biological repeats on separate occasions). Top and bottom dotted lines show the standard errors of the means. Growth of the host bacteria was taken as the positive control, and the growth medium (LB) was used as the negative control.

Furthermore, to examine the rate of bacterial killing by phages, we performed growth kill assays. The three sublines of T4 were mixed at a multiplicity of infection (MOI) of 0.01 with actively growing (optical density at 600 nm [OD_600_] ∼ 0.2) E. coli B and K-12, and growth was measured by determining OD_600_ at 5-min intervals for 16 h. All three sublines of T4 were able to suppress the growth of E. coli B and E. coli K-12 within 1 h, with a sharp reduction in OD observed in E. coli K-12 with all three T4 sublines (Fig. 5). The T4 sublines showed different growth patterns between the two strains of E. coli, but there were no significant differences between the growth patterns of the T4 sublines.

Finally, we examined the host range of the T4 sublines on a selection of laboratory and pathogenic strains of E. coli. We used three standard laboratory strains and eight pathogenic strains for this assay. On these spot plate assays, we observed that T4 phages had lytic activity against 6 of 11 strains, but there was no difference in host range between the T4 sublines (Table 4).

**TABLE 4:** Host range of T4 sublines on laboratory and clinical strains of E. coli

E. coli strain	Lysis on host^*a*^		
	T4-Hancock	T4-Rao	T4-Barr
FADDI-EC011	+	+	+
FADDI-EC012	−	−	−
FADDI-EC013	−	−	−
FADDI-EC026	−	−	−
FADDI-EC029	−	−	−
EPEC E2348/69	−	−	−
ETEC ND754	+	+	+
ETEC ND780	+	+	+
BL21 (DE3)	+	+	+
TOP 10	+	+	+
DH5a	+	+	+

a +, lysis in spot assay; −, no lysis in spot assay.

## DISCUSSION

T-series phages have been used as model systems to understand the fundamental molecular biology of life since the 1940s (3, 5, 6, 24–27). Scientists throughout the world have been investigating the genetics and physiology of these bacteriophages, with many of the subsequent discoveries laying the foundation for the entire phage field. T4 phages in particular have been used as model organisms in systems biology to understand important biological phenomena, including the bacteriophage adherence to mucus (BAM) model, which was used to unravel tripartite symbioses between phages, microbes, and the gut mucosa (28), and *in vivo* studies to understand phage-bacterial dynamics in the gut (29). Given that these model phages have been distributed, maintained, and propagated across many different laboratories in a multitude of ways, there exists the possibility for phenotypic and genotypic differences to accumulate between strains from different laboratories, which could ultimately compromise the comparability of these important model organisms.

To address this, we investigated the genetic and phenotypic changes between a 48-year-old T-series phage stock and contemporary laboratory strains. Our analysis of three T4 sublines revealed minor genetic differences and no detectable variation in growth characteristic in their usual hosts, E. coli B and E. coli K-12. We did, however, observe substantial differences between the NCBI RefSeq nucleotide sequence (accession no. AF158101) and those of our three T4 sublines, highlighting the need for an updated reference genome for T4 phage.

The complete genome of T4 was initially established by sequencing small fragments that were obtained following cloning and direct PCR (15). According to the GenBank record, the first information on T4's genome sequence was recorded in 1981 and its latest update was listed in 2003 (AF158101.6). A relatively high genetic variation between the GenBank RefSeq entry and T4 sublines of this study indicates that there may be artifacts in the original GenBank reference sequence. We therefore propose T4-Hancock as an updated reference genome for the field, and we used this genome as the reference in our analysis. The complete genome of T4-Hancock was 5 bp longer than the T4 RefSeq sequence. This difference may have arisen due to a surprisingly high number of nucleotide variations between these two genomes. The difference in the genome length may also be related to the fact that the genome of T4 is terminally redundant and circularly permuted, potentially altering the total length of chromosome between generations (30). Unlike T4, we observed less variability in T2- and T6-Hancock compared to NCBI RefSeq data. The quality of NCBI RefSeq may be the main reason for this. NCBI database record shows that T2 (nucleotide accession no. LC348380) and T6 (nucleotide accession no. MH550421) genomes were completed using Illumina and uploaded in 2018. Furthermore, this also corresponds with our finding of less variability in T4-Barr and T4-Rao genomes compared with T4-Hancock.

We further examined the genetic divergence between historical T4 and two contemporary stocks of T4 (T4-Barr and T4-Rao). Our analysis showed the insertion of 13 bp in T4-Barr and 14 bp in T4-Rao compared with T4-Hancock. This finding is in line with the total chromosome length of each subline that we obtained in our study. The average nucleotide identity, although the difference was very small, showed that the two contemporary sublines, T4-Barr and T4-Rao, shared a higher percentage of similarity to each other than they shared with T4-Hancock. Interestingly, both contemporary sublines also shared the nucleotide variations, with T4-Rao having five additional variations. The majority of nucleotide variations were in essential genes, including genes that encode long tail fibers (gp34 and gp37), tail sheath (gp7), and enzymes associated with DNA metabolism. Although we could not track the history of our contemporary T4 phages, it is likely that these phages have gone through indefinite rounds of propagation, purification, and enumeration on various E. coli strains, the results of which may be associated with bottlenecks and mutational drift (17). Furthermore, the difference in mutation rate between T4-Rao and T4-Barr indicates that the mutational drift in T4 phages might have been driven by the different scale of work conducted between the laboratories, highlighting the possibility of genetic diversity of T4 sublines between laboratories across the world.

However, we did not observe any noticeable differences in the growth characteristics between T4 sublines in our experimental conditions. Nevertheless, mutations in the essential structural genes, such as the long tail fibers, could potentially affect the phage’s adsorption to its host. T4 phage uses the C-terminal region of the distal subunit (gp37) of the long tail fibers to recognize host cellular receptors (31, 32), and it has been shown that mutations in this region, which comprises about 140 amino acid residues in a 1,026-residue-long polypeptide, could expand the host range of T4 and T4-like phages (33, 34). Our analysis showed that T4-Rao had a missense mutation in amino acid residue 417 of gp37, but we could not find any differences in host range between the T4 sublines on examination against 11 different laboratory and pathogenic strains of E. coli.

This study also examined the stability of T-series phages in prolonged storage. All the T-even series lysates had active phages present, which could be propagated in laboratory after 48 years in storage. However, we were not able to recover any viable phages from the lysate of T-odd series (T3 and T7). We do not have complete information on the long-term storage conditions of these phages over the last ∼50 years. However, all T-series stocks were stored in the same box and therefore under the same conditions. Furthermore, initial titers of the stock, as labeled on the vials, were also broadly similar between T-odd and T-even phages. Therefore, our results suggest that the T-even series could be more stable than the T-odd series on prolonged storage. This finding is further supported by the close genetic relatedness of T-even phages, compared with the diversity seen across the T-odd phages (11). However, evidence from studies of T-odd phages stored at different time frame is required to verify this finding.

More recently, there has been rising concern regarding the reproducibility of scientific research. The inability to identify or use suitable model organisms is a major problem for reproducibility (35). Given that the variation could be possible in T4 sublines, phage researchers should be vigilant in their use and propagation of these model bacteriophages. For example, a study that investigated T7 and T3 sublines from different sources showed a high variability between sublines across laboratories, some of which were found to be using entirely different strains (36). Our *in silico* analysis of T4-like genomes further supports this, providing evidence that there were large variations in genome sequences between T4-like phages. However, some of the T4-like strains, although named differently in the NCBI database, closely matched the wild-type T4 strain.

In conclusion, our analyses suggest that individually maintained T4 sublines undergo continuous genetic drift that may cause microevolution in the model bacteriophage stocks. The genetic variation in T4 sublines did not show a difference in our phenotypic analysis, which is a favorable finding for the phage community, and the rationale proposed by Delbruck and coworkers on the use of model bacteriophages to avoid incomparability of results between laboratories proved to be still valid (37). However, the magnitude of genetic changes may vary between laboratories, which highlights a need for a larger-scale comparative study of model bacteriophages sublines.

## MATERIALS AND METHODS

### Bacteriophage stock.

The stock of T-series bacteriophages in this study was obtained from Peter Reeves (University of Sydney, Australia). The stock comprised lysates of T1, T2, T3, T4, T6, and T7 phages, purified and stored in sealed glass ampules with chloroform in 1972. These phages were used in several studies by Robert E. Hancock in the 1970s (referred to as T#-Hancock here) (13, 14, 38, 39). T1 phage was not used in this study due to its potential contamination hazard for our laboratory. These stocks have been stored in a cold room (∼4°C) since the 1970s. In addition, we used two contemporary strains of T4; received from the Bacteriophage T4 lab at the Catholic University of America, led by Venigalla B. Rao (called T4-Rao here) and the phage collection of our lab (called T4-Barr here). T4-Rao was originally received from August H. Doermann, University of Washington, before 1990 (V. B. Rao, personal communication). T4-Barr was originally purchased from the ATCC by Forest Rohwer, San Diego State University, where it had been used for more than 5 years before being acquired by the Barr lab in 2016.

### Bacterial strains.

E. coli B (ATCC 11303), E. coli K-12 (ATCC 25922), and E. coli BL21(DE3) were used as hosts to propagate T-series phages. In addition, 10 other E. coli strains were used in host range assays: five strains (FADDI-EC011, -12, -13, -26, and -29) which were isolated from various clinical samples in Australia and were received from Jian Li’s laboratory, Monash University, Australia; one standard laboratory pathogenic strain, enteropathogenic *E. coli* (EPEC) strain E2348/69, described previously (40); two pathogenetic strains (enterotoxigenic *E. coli* [ETEC] strains ND754 and ND780) received from Roy M. Robins-Browne, University of Melbourne, Australia; and two commercial E. coli laboratory strains (TOP10 [Thermo Fisher Scientific, USA; C4040-10] and DH5a [New England Biolabs, USA; C2987H]). All pathogenic strains were collected from institutional repositories without identifiable patient data, and all experiments followed institutional guidelines.

### Bacteriophage revival and propagation.

Lysates from the old stocks were each recovered in 15-ml Falcon tubes. One milliliter of lysate was mixed with susceptible hosts (E. coli B for T2, -3, -4, and -6 and E. coli BL21 for T7) and plated onto lysogeny broth (LB) agar plates using the soft agar overlay method, followed by incubation for 18 to 24 h at 37°C. Each plating was done in duplicate and repeated with lysates from at least three different vials. In instances of no plaque formation, amplification was attempted using the original lysate and its host in broth culture, which was then replated. Lysates with viable phages were propagated following the Phage-on-Tap protocol (41).

### Phage DNA extraction and sequencing.

High-titer lysate (>10^9^ PFU/ml) was used for DNA extraction. To remove host nucleic acid contamination, lysates was treated with DNase (1 mg/ml) and RNase A (12.5 mg/ml) for 2 h at 37°C, followed by an inactivation step of 5 min at 75°C. DNA was extracted using Norgen phage DNA extraction kit (Norgen Biotek, Ontario, Canada), following the manufacturer’s instructions. The extracted DNA was vacuum dried into a pellet for transport. Sequencing was performed using the Illumina HiSeq 150-bp paired-end platform at the Genewiz facilities (Suzhou, China).

### Bioinformatics analysis.

Illumina HiSeq platform generated five to six million raw reads from each sample. Trimmomatic v0.39 (42) was used to remove adaptor sequences and to truncate reads with a quality score less than 15 in a sliding window of 4 bp. Raw reads were assembled using Unicycler v0.43 (43) with a flag for minimum contig length of 1,000 bp, which produced a single and complete genome. We aligned NCBI RefSeq sequences (T2 nucleotide accession no. LC348380; T4 nucleotide accession no. AF158101; T6 nucleotide accession no. MH550421) with the assembled genomes using Mauve genome alignment (44), and the results from the alignment were used to rearrange nucleotides to match the start position of NCBI RefSeq entries. The genomes were then annotated manually; copying the annotation from their respective NCBI RefSeq entries using Geneious v 9.1.8 (45), provided a sequence similarity for the coding region of at least 98%, followed by manual curation where required.

To identify nucleotide variations, the filtered raw reads were mapped to either NCBI RefSeq entries or our assembled genomes using Snippy v4.2 (https://github.com/tseemann/snippy) with the setting of the minimum number of reads covering a site to be considered at 100 and the minimum variant call format (VCF) variant call quality also at 100. To compare nucleotide identity, the complete genomes were aligned by pairwise sequence alignment as implemented in Pyani (https://huttonics.github.io/pyani/). The average nucleotide identity (ANI), as a percentage, obtained from the analysis was converted into a matrix and visualized using pheatmap (46) in R package.

### Determination of T4 growth characteristics.

The growth characteristics of all three T4 phages were examined using the hosts E. coli B and E. coli K-12. First, relative efficiency of platting between hosts was examined. Each lysate was serially diluted to a titer of ∼100 PFU/ml, and 500 μl was used in a soft-agar overlay with each host. After overnight growth, PFU were counted and recorded, and the relative efficiency of plating on E. coli K-12 was calculated as the ratio of PFU in E. coli K-12 to PFU in E. coli B. Each experiment was performed in duplicate and repeated three times.

Next, one-step growth curve experiments were performed on E. coli B and E. coli K-12 separately with each T4 subline. Bacteria from overnight broth cultures were diluted 1:20 in LB and allowed to grow until an optical density 600 nm (OD_600_) of 0.2 was reached (∼2 h), and the culture was then infected with phage at a multiplicity of infection (MOI) of 0.1. Phage was allowed to adsorb for 5 min at 37°C with orbital agitation at 120 rpm. The mixture was then pelleted (4,000 × *g*, 2 min, room temperature), resuspended in fresh LB broth, and incubated at 37°C and 120 rpm. Samples (100 μl) were repeatedly taken every 5 or 10 min for a total period of 1 h, transferred into chloroform-saturated phosphate-buffered saline (PBS), serially diluted, and plated to determine numbers of PFU. The number of bacteria in the initial inoculum was determined by plate count. The number of PFU per infected cell was calculated by dividing PFU by initial density of bacteria. The experiment was repeated on at least three different occasions.

Bacterial lysis by phage was determined from sequential OD readings obtained using a microtiter plate reader. Bacterial cultures at exponential growth were obtained as described above. Each culture was added to individual wells of a 96-well microtiter plate and mixed with phage lysate at an MOI of 0.01. The plate was incubated at 37°C in a microplate reader (Epoch Microplate spectrophotometer; BioTek, Winooski, VT, USA) with continuous shaking, and the data were recorded every 5 min.

To gauge the host range of each T4 subline, we used 11 different clinical and laboratory isolates of E. coli and the standard spot plate assay (47). High-titer (>10^10^ PFU/ml) phage lysate (10 μl) was spotted on the surface of agar plate seeded with host bacteria. The zone of clearance on the spotted area was recorded as a positive lytic activity.

### Graphing, data presentation, and statistical analysis.

All data were analyzed and visualized using GraphPad Prism v8. Averages and standard deviations for all the replicates were calculated and compared using a *t* test to obtain *P* values. To infer statistical significance, the threshold was set at *P* values of <0.05.

### Data availability.

The nucleotide sequence of T4-Hancock is available in NCBI GenBank under accession number MT984581. Raw sequence data and alignment files are available in NCBI BioProject under ID PRJNA662192.

## Supplementary Material

Reviewer comments

## References

[1] Rohwer F, Segall AM. 2015. In retrospect: a century of phage lessons. Nature 528:46–48. doi:10.1038/528046a. 26632584

[2] Salmond GP, Fineran PC. 2015. A century of the phage: past, present and future. Nat Rev Microbiol 13:777–786. doi:10.1038/nrmicro3564. 26548913

[3] Luria SE, Delbruck M. 1943. Mutations of bacteria from virus sensitivity to virus resistance. Genetics 28:491–511. 17247100 10.1093/genetics/28.6.491 PMC1209226

[4] Luria SE, Human ML. 1952. A nonhereditary, host-induced variation of bacterial viruses. J Bacteriol 64:557–569. doi:10.1128/JB.64.4.557-569.1952. 12999684 PMC169391

[5] Crick FH, Barnett L, Brenner S, Watts-Tobin RJ. 1961. General nature of the genetic code for proteins. Nature 192:1227–1232. doi:10.1038/1921227a0. 13882203

[6] Sanger F, Air GM, Barrell BG, Brown NL, Coulson AR, Fiddes CA, Hutchison CA, Slocombe PM, Smith M. 1977. Nucleotide sequence of bacteriophage phi X174 DNA. Nature 265:687–695. doi:10.1038/265687a0. 870828

[7] Barrangou R, Fremaux C, Deveau H, Richards M, Boyaval P, Moineau S, Romero DA, Horvath P. 2007. CRISPR provides acquired resistance against viruses in prokaryotes. Science 315:1709–1712. doi:10.1126/science.1138140. 17379808

[8] Doudna JA, Charpentier E. 2014. Genome editing. The new frontier of genome engineering with CRISPR-Cas9. Science 346:1258096. doi:10.1126/science.1258096. 25430774

[9] Gordillo Altamirano FL, Barr JJ. 2019. Phage therapy in the postantibiotic era. Clin Microbiol Rev 32:e00066-18. doi:10.1128/CMR.00066-18. 30651225 PMC6431132

[10] Demerec M, Fano U. 1945. Bacteriophage-resistant mutants in *Escherichia coli*. Genetics 30:119–136. 17247150 10.1093/genetics/30.2.119 PMC1209279

[11] Abedon ST. 2000. The murky origin of Snow White and her T-even dwarfs. Genetics 155:481–486. 10835374 10.1093/genetics/155.2.481 PMC1461100

[12] Kutter EM, Wiberg JS. 1968. Degradation of cytosin-containing bacterial and bacteriophage DNA after infection of *Escherichia coli* B with bacteriophage T4D wild type and with mutants defective in genes 46, 47 and 56. J Mol Biol 38:395–411. doi:10.1016/0022-2836(68)90394-x. 4305016

[13] Hancock RE, Reeves P. 1975. Bacteriophage resistance in *Escherichia coli* K-12: general pattern of resistance. J Bacteriol 121:983–993. doi:10.1128/JB.121.3.983-993.1975. 1090611 PMC246027

[14] Hancock RE, Davies JK, Reeves P. 1976. Cross-resistance between bacteriophages and colicins in *Escherichia coli* K-12. J Bacteriol 126:1347–1350. doi:10.1128/JB.126.3.1347-1350.1976. 780346 PMC233163

[15] Miller ES, Kutter E, Mosig G, Arisaka F, Kunisawa T, Ruger W. 2003. Bacteriophage T4 genome. Microbiol Mol Biol Rev 67:86–156. doi:10.1128/mmbr.67.1.86-156.2003. 12626685 PMC150520

[16] Dharmalingam K, Revel HR, Goldberg EB. 1982. Physical mapping and cloning of bacteriophage T4 anti-restriction endonuclease gene. J Bacteriol 149:694–699. doi:10.1128/JB.149.2.694-699.1982. 6276366 PMC216561

[17] Petrov VM, Ratnayaka S, Nolan JM, Miller ES, Karam JD. 2010. Genomes of the T4-related bacteriophages as windows on microbial genome evolution. Virol J 7:292. doi:10.1186/1743-422X-7-292. 21029436 PMC2993671

[18] Bryson AL, Hwang Y, Sherrill-Mix S, Wu GD, Lewis JD, Black L, Clark TA, Bushman FD. 2015. Covalent modification of bacteriophage T4 DNA inhibits CRISPR-Cas9. mBio 6:e00648-15. doi:10.1128/mBio.00648-15. 26081634 PMC4471564

[19] Yaung SJ, Esvelt KM, Church GM. 2015. Complete genome sequences of T4-like bacteriophages RB3, RB5, RB6, RB7, RB9, RB10, RB27, RB33, RB55, RB59, and RB68. Genome Announc 3:e01122-14. doi:10.1128/genomeA.01122-14. PMC4293622 25555735

[20] Chibani-Chennoufi S, Canchaya C, Bruttin A, Brüssow H. 2004. Comparative genomics of the T4-Like *Escherichia coli* phage JS98: implications for the evolution of T4 phages. J Bacteriol 186:8276–8286. doi:10.1128/JB.186.24.8276-8286.2004. 15576776 PMC532421

[21] Washizaki A, Yonesaki T, Otsuka Y. 2016. Characterization of the interactions between Escherichia coli receptors, LPS and OmpC, and bacteriophage T4 long tail fibers. Microbiologyopen 5:1003–1015. doi:10.1002/mbo3.384. 27273222 PMC5221442

[22] Yu F, Mizushima S. 1982. Roles of lipopolysaccharide and outer membrane protein OmpC of Escherichia coli K-12 in the receptor function for bacteriophage T4. J Bacteriol 151:718–722. doi:10.1128/JB.151.2.718-722.1982. 7047495 PMC220313

[23] Ellis EL, Delbruck M. 1939. The growth of bacteriophage. J Gen Physiol 22:365–384. doi:10.1085/jgp.22.3.365. 19873108 PMC2141994

[24] Benzer S. 1955. Fine structure of a genetic region in bacteriophage. Proc Natl Acad Sci U S A 41:344–354. doi:10.1073/pnas.41.6.344. 16589677 PMC528093

[25] Jacob F, Monod J. 1961. Genetic regulatory mechanisms in the synthesis of proteins. J Mol Biol 3:318–356. doi:10.1016/s0022-2836(61)80072-7. 13718526

[26] Smith HO, Hutchison CA, III, Pfannkoch C, Venter JC. 2003. Generating a synthetic genome by whole genome assembly: phiX174 bacteriophage from synthetic oligonucleotides. Proc Natl Acad Sci U S A 100:15440–15445. doi:10.1073/pnas.2237126100. 14657399 PMC307586

[27] Rath D, Amlinger L, Rath A, Lundgren M. 2015. The CRISPR-Cas immune system: biology, mechanisms and applications. Biochimie 117:119–128. doi:10.1016/j.biochi.2015.03.025. 25868999

[28] Barr JJ, Auro R, Furlan M, Whiteson KL, Erb ML, Pogliano J, Stotland A, Wolkowicz R, Cutting AS, Doran KS, Salamon P, Youle M, Rohwer F. 2013. Bacteriophage adhering to mucus provide a non-host-derived immunity. Proc Natl Acad Sci U S A 110:10771–10776. doi:10.1073/pnas.1305923110. 23690590 PMC3696810

[29] Lourenço M, Chaffringeon L, Lamy-Besnier Q, Pédron T, Campagne P, Eberl C, Bérard M, Stecher B, Debarbieux L, De Sordi L. 2020. The spatial heterogeneity of the gut limits predation and fosters coexistence of bacteria and bacteriophages. Cell Host Microbe 28:390–401.E5. doi:10.1016/j.chom.2020.06.002. 32615090

[30] Streisinger G, Emrich J, Stahl MM. 1967. Chromosome structure in phage t4, iii. Terminal redundancy and length determination. Proc Natl Acad Sci U S A 57:292–295. doi:10.1073/pnas.57.2.292. 16591467 PMC335503

[31] Montag D, Riede I, Eschbach ML, Degen M, Henning U. 1987. Receptor-recognizing proteins of T-even type bacteriophages. Constant and hypervariable regions and an unusual case of evolution. J Mol Biol 196:165–174. doi:10.1016/0022-2836(87)90519-5. 2958637

[32] Montag D, Hashemolhosseini S, Henning U. 1990. Receptor-recognizing proteins of T-even type bacteriophages. The receptor-recognizing area of proteins 37 of phages T4 TuIa and TuIb. J Mol Biol 216:327–334. doi:10.1016/S0022-2836(05)80324-9. 2147721

[33] Tétart F, Repoila F, Monod C, Krisch HM. 1996. Bacteriophage T4 host range is expanded by duplications of a small domain of the tail fiber adhesin. J Mol Biol 258:726–731. doi:10.1006/jmbi.1996.0281. 8637004

[34] Chen M, Zhang L, Abdelgader SA, Yu L, Xu J, Yao H, Lu C, Zhang W. 2017. Alterations in gp37 expand the host range of a T4-like phage. Appl Environ Microbiol 83:e01576-17. doi:10.1128/AEM.01576-17. 28939606 PMC5691408

[35] Vasilevsky NA, Brush MH, Paddock H, Ponting L, Tripathy SJ, Larocca GM, Haendel MA. 2013. On the reproducibility of science: unique identification of research resources in the biomedical literature. PeerJ 1:e148. doi:10.7717/peerj.148. 24032093 PMC3771067

[36] Studier FW. 1979. Relationships among different strains of T7 and among T7-related bacteriophages. Virology 95:70–84. doi:10.1016/0042-6822(79)90402-1. 375582

[37] Anderson TF. 1992. Electron microscopy of phages, p 336. *In* Cairns J, Stent GS, Watson JD (ed), Phage and the origins of molecular biology. Cold Spring Harbor Laboratory Press, Cold Spring Harbor, NY.

[38] Hancock RW, Braun V. 1976. Nature of the energy requirement for the irreversible adsorption of bacteriophages T1 and phi80 to *Escherichia coli*. J Bacteriol 125:409–415. doi:10.1128/JB.125.2.409-415.1976. 128553 PMC236097

[39] Hancock RE, Reeves P. 1976. Lipopolysaccharide-deficient, bacteriophage-resistant mutants of *Escherichia coli* K-12. J Bacteriol 127:98–108. doi:10.1128/JB.127.1.98-108.1976. 776951 PMC233038

[40] Schmidt H, Russmann H, Karch H. 1993. Virulence determinants in nontoxinogenic *Escherichia coli* O157 strains that cause infantile diarrhea. Infect Immun 61:4894–4898. doi:10.1128/IAI.61.11.4894-4898.1993. 8406892 PMC281251

[41] Bonilla N, Rojas MI, Netto Flores Cruz G, Hung SH, Rohwer F, Barr JJ. 2016. Phage on tap—a quick and efficient protocol for the preparation of bacteriophage laboratory stocks. PeerJ 4:e2261. doi:10.7717/peerj.2261. 27547567 PMC4975003

[42] Bolger AM, Lohse M, Usadel B. 2014. Trimmomatic: a flexible trimmer for Illumina sequence data. Bioinformatics 30:2114–2120. doi:10.1093/bioinformatics/btu170. 24695404 PMC4103590

[43] Wick RR, Judd LM, Gorrie CL, Holt KE. 2017. Unicycler: resolving bacterial genome assemblies from short and long sequencing reads. PLoS Comput Biol 13:e1005595. doi:10.1371/journal.pcbi.1005595. 28594827 PMC5481147

[44] Darling AE, Mau B, Perna NT. 2010. progressiveMauve: multiple genome alignment with gene gain, loss and rearrangement. PLoS One 5:e11147. doi:10.1371/journal.pone.0011147. 20593022 PMC2892488

[45] Kearse M, Moir R, Wilson A, Stones-Havas S, Cheung M, Sturrock S, Buxton S, Cooper A, Markowitz S, Duran C, Thierer T, Ashton B, Meintjes P, Drummond A. 2012. Geneious Basic: an integrated and extendable desktop software platform for the organization and analysis of sequence data. Bioinformatics 28:1647–1649. doi:10.1093/bioinformatics/bts199. 22543367 PMC3371832

[46] Kolde R. 2012. Pheatmap: pretty heatmaps. R package version 1.

[47] Kutter E. 2009. Phage host range and efficiency of plating. Methods Mol Biol 501:141–149. doi:10.1007/978-1-60327-164-6_14. 19066818

