# Supplementary material for: Temporal Stability and Genetic Diversity of 48-Year-Old T-Series Phages
Source: mSystems. 2021 Feb 16;6(1):e00990-20. doi: 10.1128/mSystems.00990-20 (PMC8573959; doi:10.1128/mSystems.00990-20)
Supplement: FIG S1 [file msystems.00990-20-sf001.pdf]

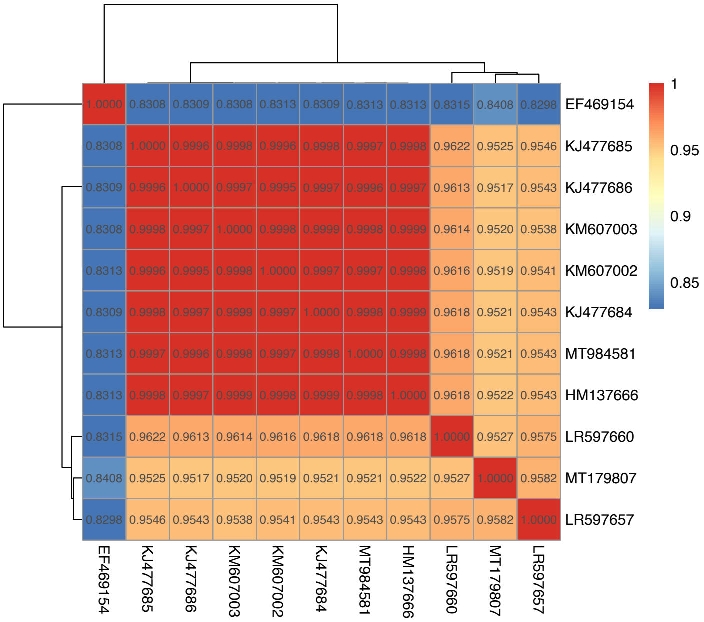

Accession number	GenBank Entry Title	Reference
HM137666	Enterobacteria phage T4T, complete genome	(Petrov et al 2010)
KJ477684	Enterobacteria phage T4 strain wild, complete genome	(Bryson et al 2015)
KJ477685	Enterobacteria phage T4 strain 147, complete genome	(Bryson et al 2015)
KJ477686	Enterobacteria phage T4 strain GT7, complete genome	(Bryson et al 2015)
KM607002	Enterobacteria phage RB55, complete genome	(Yaung et al 2015)
KM607003	Enterobacteria phage RB59, complete genome	(Yaung et al 2015)
EF469154	Enterobacteria phage JS98, complete genome	(Chibani-Chennoufi et al 2004)
LR597660	Escherichia phage T4_ev151 genome assembly, chromosome: 1	NA
MT179807	Escherichia phage vB_EcoM_IME537, complete genome	NA
LR597657	Escherichia phage T4_ev240 genome assembly, chromosome: 1	NA

References:

Bryson AL, Hwang Y, Sherrill-Mix S, Wu GD, Lewis JD, Black L *et al* (2015). Covalent Modification of Bacteriophage T4 DNA Inhibits CRISPR-Cas9. *mBio* **6:** e00648.

Chibani-Chennoufi S, Canchaya C, Bruttin A, Brüssow H (2004). Comparative genomics of the T4-Like Escherichia coli phage JS98: implications for the evolution of T4 phages. *Journal of bacteriology* **186:** 8276-8286.

Petrov VM, Ratnayaka S, Nolan JM, Miller ES, Karam JD (2010). Genomes of the T4-related bacteriophages as windows on microbial genome evolution. *Virology journal* **7:** 292.

Yaung SJ, Esvelt KM, Church GM (2015). Complete Genome Sequences of T4-Like Bacteriophages RB3, RB5, RB6, RB7, RB9, RB10, RB27, RB33, RB55, RB59, and RB68. *Genome announcements* **3**.
