# Supplementary material for: Temporal Stability and Genetic Diversity of 48-Year-Old T-Series Phages
Source: mSystems. 2021 Feb 16;6(1):e00990-20. doi: 10.1128/mSystems.00990-20 (PMC8573959; doi:10.1128/mSystems.00990-20)
Supplement: Reviewer comments [file reviewer-comments.pdf]

AMERICAN SOCIETY FOR MICROBIOLOGY | mSystems®

Genomic and chemical diversity of *Bacillus subtilis* secondary metabolites against plant pathogenic fungi

Heiko T Kiesewalter, Carlos N Lozano-Andrade, Mario Wibowo, Mikael Strube, Gergely Maróti, Daniel Snyder, Tue Sparholt Jørgensen, Thomas Larsen, Vaughn Cooper, Tilmann Weber, and Ákos T Kovács

Corresponding Author(s): Ákos T Kovács, Technical University of Denmark

Review Timeline:

Submission Date:	August 6, 2020
Editorial Decision:	October 7, 2020
Revision Received:	January 21, 2021
Accepted:	January 27, 2021

Editor: Matthew Traxler

Reviewer(s): Disclosure of reviewer identity is with reference to reviewer comments included in decision letter(s). The following individuals involved in review of your submission have agreed to reveal their identity: Joachim Vater (Reviewer #3)

Transaction Report:

(Note: With the exception of the correction of typographical or spelling errors that could be a source of ambiguity, letters and reports are not edited. The original formatting of letters and referee reports may not be reflected in this compilation.)

DOI: https://doi.org/10.1128/mSystems.00770-20

September 28, 2020

Prof. Ákos T Kovács
Technical University of Denmark
Department of Biotechnology and Biomedicine
Søltofts Plads 221
Kgs Lyngby 2800
Denmark

Re: mSystems00770-20 (Genomic and chemical diversity of *Bacillus subtilis* secondary metabolites against plant pathogenic fungi)

Dear Prof. Ákos T Kovács:

Overall, the reviewers were positive about your manuscript. Thus, I am returning it to you with a request for 'minor modification.' Please note that reviewer 1 raised concerns surrounding the possible synergism of surfactin and plipistatin. I would ask that your revisions address these concerns specifically, as well as the remainder of the points raised by all reviewers.

Below you will find the comments of the reviewers.

To submit your modified manuscript, log onto the eJP submission site at https://msystems.msubmit.net/cgi-bin/main.plex. If you cannot remember your password, click the "Can't remember your password?" link and follow the instructions on the screen. Go to Author Tasks and click the appropriate manuscript title to begin the resubmission process. The information that you entered when you first submitted the paper will be displayed. Please update the information as necessary. Provide (1) point-by-point responses to the issues raised by the reviewers as file type "Response to Reviewers," not in your cover letter, and (2) a PDF file that indicates the changes from the original submission (by highlighting or underlining the changes) as file type "Marked Up Manuscript - For Review Only."

Due to the SARS-CoV-2 pandemic, our typical 60 day deadline for revisions will not be applied. I hope that you will be able to submit a revised manuscript soon, but want to reassure you that the journal will be flexible in terms of timing, particularly if experimental revisions are needed. When you are ready to resubmit, please know that our staff and Editors are working remotely and handling submissions without delay. If you do not wish to modify the manuscript and prefer to submit it to another journal, please notify me of your decision immediately so that the manuscript may be formally withdrawn from consideration by mSystems.

If your manuscript is accepted for publication, you will be contacted separately about payment when the proofs are issued; please follow the instructions in that e-mail. Arrangements for payment must be made before your article is published. For a complete list of **Publication Fees**, including supplemental material costs, please visit our website.

Corresponding authors may join or renew ASM membership to obtain discounts on publication fees. Need to upgrade your membership level? Please contact Customer Service at Service@asmusa.org.

Thank you for submitting your paper to mSystems.

Sincerely,

Matthew Traxler

Editor, mSystems

Journals Department
American Society for Microbiology
1752 N St., NW
Washington, DC 20036
E-mail: peerreview@asmusa.org
Phone: 1-202-942-9338

Reviewer comments:

Reviewer #1 (Comments for the Author):

In this manuscript, Kovács et al would like to display the genomic and chemical diversity of Bacillus subtilis secondary metabolites against plant pathogenic fungi, by detailed comparison of the antagonistic effect of B. subtilis WT isolates and their NRPS deletion mutants against Fusarium and Botrytis, and further evaluation of two lipopeptides (surfactin and plipastatin) production, and prediction of secondary metabolite BGC by antiSMASH. The authors show the different antifungal capacity of B. subtilis correlates to the production of antifungal lipopeptides (surfactin and plipastatin) and their BGC content, and explain the genetic details of loss of the production of lipopeptide is based on nonsense mutation on the regulator process and lack of core biosynthesis genes. Besides the interspecies interaction, intraspecies interaction among these isolates is investigated and assumed to correlate to the BGC prediction result. Instead of single strain analysis, this manuscript shows that the similar B. subtilis can exhibit different antifungal capacity, based on the different production of potential antifungal lipopeptides and corresponding BGC content. This knowledge will foster an understanding of the ecological role of B. subtilis and transfers knowledge to the future biocontrol application.

Overall this research is well designed and the experiments are well performed and lead to the production of reliable data for sufficient interpretation and discussion. However, in order to make this research more suitable to publish on mSystems as high-quality research and match the broader readership, please see the following suggestions:

1) In the 'testing the antifungal potential of B. subtilis isolates' section, it is better to add the antifungal activity of P5-B2 and P8-B2 in figure 1A, since the gene cluster prediction from those two strains are discussed in figure 3. The difficulty of knockout (not naturally competent) can be commented into figure legend (Page 8, Line 164-166.

2) Page 9, Line 173-Line 188: the result section regarding antifungal potential against B. cinerea is a bit confused and not easy to follow. Could be possible to group the isolates as (or something similar): sfp-dependent (most of the isolates) and partial sfp-dependent (64 and MB8_B7); under

sfp-dependent, there are plipastatin-dependent (39, P8_B1, Pb_B3) and partial plipastatin-dependent (MB8_B1, MB11_B1, MB12_B1, MB12_B3, MB12_B4, P5_B1) and synergistic effect (38, 72, 75, 77). The outlier will be 73, MB9_B4 and MB9_B6, which represent weaker antifungal from WT isolates.

3) In the section 'synergism between plipastatin and surfactin...', the statement 'the deletion mutant screen in combination with chemical profiling suggested that the presence of either plipastatin or surfactin is sufficient for inhibiting the growth of B. cinerea', however it is not enough to explain the strain 64 and MB8_B7 and a bit contradiction with the following statement. Please rephrase this paragraph (Line 211-Line 220) to make it more concise.

4) As standards of surfactin and plipastatin are available (LCMS standard), the antifungal activity of reference compounds should be tested and added to figure 1A; the same experiment should be performed for combinations of surfactin and plipastatin! These experiments will be essential to support the findings of the knockout study to clarify the synergism between plipastatin and surfactin for B. cinerea inhibition.

5) In Figure 2C, the targeted LCMS chromatograms of surfactin and plipastatin show multiple peaks, please clarify or mention whether they are isomers/analogs.

6) In Figure 2C, it seems the multiple pattern of surfactin and plipastatin from B.subtilis isolates is different from the standards (when zoomed in). Please clarify the composition or explain the differences.

7) Bacilysin is also reported as antifungal NRPS product and the BGC is widely spread in all B. subtilis isolates. Please clarify or discuss whether the antifungal activity can from bacilysin or not, althrough bacilysin is not sfp-dependent NRP.

8) In Figure 4A, please indicate the mutation of comA in MB9_B4 to show the reason of non-production of surfactin in the Figure 4A (Page 12, Lin 263). It is better to present in the main text instead of SI.

9) The section of 'intraspecies interaction' is not well interpreted and even no discussion about the results is included. The only prediction of potential BGC is not sufficient to explain the interaction assay (Figure 5).

10) Page 11, Line 224: please comment the 'in addition to one B.Licheniformis strain used as an outlier' is P8_B2).

11) Page 11, Line 226: please clarify 'all predicted BGCs' or 'all predicted peptidic BGCs'. It is better to mention whether some other gene clusters are present or not, in order to discuss the overall contribution of secondary metabolites (or their BGCs).

12) Page 5, line 99, please clarify or give full name of sfp for the first time.

13) Page 12, line 263, please clarify or give full name of comA for the first time.

Reviewer #2 (Comments for the Author):

This work provides interesting results showing that different Bacillus strains of the same subtilis species are not equal regarding their potential to produce cyclic lipopeptides as important bioactive secondary metabolites. It is quite unexpected considering the recent genomic analyses correlated with phylogeny from which it can be deduced that B. subtilis is invariabily a producer of surfactin and fengycin/plipastatin. Not easy to explain but it suggests an underestimated effect of environmental pressure in shaping the genetic basis for lipopeptide production...
The work has been seemingly well conceived and the manuscript is generally well written but I have some remarks and concerns listed below.

L70-74: I would agree with those general statements but the authors should use more recent reviews! I'm not sure that Straight et al. showed any role as signal for lipopeptides in that study...

L78-80: Taxonomy has been adapted in the recent years and additional species (velezensis, atrophaeus) have been included in the B. subtilis complex. Again, the authors should refer to more recent papers at that place such as Harwood et al that is actually used later in the Introduction (L97)!!

L110-112: an additional example of un-appropriate use of reference works with a review and biophysic studies that are not really supporting antibacterial activity at least via leakage. The authors should use research papers unambiguously describing antimicrobial activity of surfactin based on cytolytic activity when biologically relevant concentrations (low micromolar) are tested.

L119: does the distinction between plipastatin and fengycin really matter? In other words, does the very small change in structure between the two compounds (isomery of one amino acid in the middle of a 10 residue peptide) significantly impact antimicrobial potential or bioactivity in general?

L124: Add "direct" before antimicrobial properties since it's a siderophore...

L128: I don't understand the word "systematically" here

L133: why testing two different Fusarium and not another very different fungus together with Botrytis?

End of Introduction: there is not reference to the last part of the work on interactions between bacilli! Maybe the authors do not consider it as crucial in the context of this work... and I would agree! The logic for including this interaction part is not obvious.

L173-188: The authors should refer once more to the figures in this long piece of text...

Paragraph L195-209: Is it a similar growth for all strains on PDA plates? It's not easy to accurately determine but at least upon visual assessment ? Sampling was performed in the colony? In the medium around? The way and place plugs are performed in the gelified medium may be crucial... Plipastatin peak is very low in Fig 1C... Is it really possible to discriminate/classify/compare the different strains for lipopeptide production (or better production rate) in these conditions?

L219: what do you mean by "intermediates" ? Other products from incomplete synthesis that are antifungal? It would be surprising for that kind of compound when we know the importance of a "mature" structure with entire and cyclized peptide linked to the fatty acid for bioactivity...

L233: what is the reference?

L325-326: Again, the references used do not really support the statements: a review, a biophysic paper (... on fengycin, does it work the same as plipastatin?) and the quite old paper which described the phenomenon (phospholipase inhibition) but never supported or confirmed by other studies later on...

L333: "potential intermediates": knowing the gene that was disrupted and according to the co-linearity rule, those possible intermediate products may be predicted no? What is the predicted structure? size? still cyclic?

L335-359: not useful!

L379: "ambiguous": what does it mean, no real roles? contradictory with previous statements placed several times in the manuscript...

What about the presence of BGCs (presumably silent under lab conditions) coding for undescribed NRPS products with possible antifungal activity? No prediction with Antismash?

Reviewer #3 (Comments for the Author):

In their work the authors have isolated 22 B. subtilis strains from various locations in Germany and Denmark for investigating their inhibition potential against the plant pathogenic fungi Fusarium oxysporum and graminearum as well as Botrytis cinerea. The antifungal potential of these organisms mainly depends on the action of their lipopeptide products plipastatin and surfactin. For prediction of the biocontrol effects of the B. subtilis isolates mass spectrometric product detection, mutational analysis with sfp as well as srf and pps defect mutants and genome analysis of the biosynthetic gene clusters were combined. It was shown that plipastatin efficiently inhibited the Fusarium strains, while both plipastatin and surfactin contribute to full inactivation of Botrytis cinerea.
13 of the B. subtilis isolates were selected for genome sequencing. Their potential for the production of bioactive compounds was derived from AntiSMASH 5.0 analysis specifying the biosynthetic gene clusters detected in the genomes of these organisms, but the products that are ultimately formed, can only be determined by mass spectrometric and chemical analysis. In this context the authors should comment which of the compounds listed in Fig. 3, as for example, subtilosin A, subtilomycin, sublancin, subtilin and butirosin are really expressed. Evaluation of the pps gene clusters in the genomes of plipastatin nonproducer strains revealed the reduction or complete loss of pps genes. The authors argue that in the case of BGC mutants potential intermediates might be produced that affect fungal growth. Did the authors attempt to detect, purify and characterize such intermediates?
The following figures and their legends should be modified:
Fig. 1B Please, specify SM production in the legend.
Figs. 2C; S3 and S4C The numbers and designation at the coordinates are too small. In particular, the titles at the abscissa are difficult to read.
Fig. S2 Please, specify in the legend the positions of the single NRP mutant in the middle part of this figure.

Fig. S3 Delete iturins in the legend, because they are not products of B. subtilis.
Fig. 4 The deficiency of surfactin formation by MB9_B4 (-) should be explained in the legend.
Fig. 5A is difficult to understand and should be better explained in the legend.
Fig. S5 Is MB9_B6 in the legend correct? For my opinion it should be replaced by MB9_B4 !
Amendments in the text:
Page 8, line 146: A library of 22 B. subtilis isolates....
Page 10, line 200 Where is Fig. 1C? It does not exist.
Page 10, line 201 Fig. 2B is not correct? Surfactin and plipastatin were detected in Fig. 2C!
Page 16, line 318 microorganisms
Page 17, line 350 extent
Page 17, line 343 better: model strains have rapidly lost their ability.....
Page 23, line 483 intermicrobial
Page 24, line 508 peptidyl carrier protein

In their work the authors have isolated 22 *B. subtilis* strains from various locations in Germany and Denmark for investigating their inhibition potential against the plant pathogenic fungi *Fusarium oxysporum* and *graminearum* as well as *Botrytis cinerea*. The antifungal potential of these organisms mainly depends on the action of their lipopeptide products plipastatin and surfactin. For prediction of the biocontrol effects of the *B. subtilis* isolates mass spectrometric product detection, mutational analysis with *sfp* as well as *srf* and *pps* defect mutants and genome analysis of the biosynthetic gene clusters were combined. It was shown that plipastatin efficiently inhibited the *Fusarium* strains, while both plipastatin and surfactin contribute to full inactivation of *Botrytis cinerea*.

13 of the *B. subtilis* isolates were selected for genome sequencing. Their potential for the production of bioactive compounds was derived from AntiSMASH 5.0 analysis specifying the biosynthetic gene clusters detected in the genomes of these organisms, but the products that are ultimately formed, can only be determined by mass spectrometric and chemical analysis. In this context the authors should comment which of the compounds listed in Fig. 3, as for example, subtilosin A, subtilomycin, sublancin, subtilin and butirosin are really expressed. Evaluation of the *pps* gene clusters in the genomes of plipastatin nonproducer strains revealed the reduction or complete loss of *pps* genes. The authors argue that in the case of BGC mutants potential intermediates might be produced that affect fungal growth. Did the authors attempt to detect, purify and characterize such intermediates?

The work is well-done. The results are sound and concise, but the manuscript requires revision as far as style and presentation are concerned. In particular, some of the figures and their legends should be modified:

Fig. 1B	Please, specify SM production in the legend.
Figs. 2C; S3 and S4C	The numbers and designation at the coordinates are too small. In particular, the titles at the abscissa are difficult to read.
Fig. S2	Please, specify in the legend the positions of the single NRP mutant in the middle part of this figure.
Fig. S3	Delete iturins in the legend, because they are not products of *B. subtilis*.
Fig. 4	The deficiency of surfactin formation by MB9_B4 (-) should be explained in the legend.
Fig. 5A	is difficult to understand and should be better explained in the legend.
Fig. S5	Is MB9_B6 in the legend correct? For my opinion it should be replaced by MB9_B4 !

Amendments in the text:

Page 8, line 146:	A library of 22 *B. subtilis* isolates….
Page 10, line 200	Where is Fig. 1C? It does not exist.
Page 10, line 201	Fig. 2B is not correct? Surfactin and plipastatin were detected in Fig. 2C!
Page 16, line 318	microorganisms
Page 17, line 350	extent
Page 17, line 343	better: model strains have rapidly lost their ability…..
Page 23, line 483	intermicrobial
Page 24, line 508	peptidyl carrier protein

Reviewer comments:

Reviewer #1 (Comments for the Author):

In this manuscript, Kovács et al would like to display the genomic and chemical diversity of Bacillus subtilis secondary metabolites against plant pathogenic fungi, by detailed comparison of the antagonistic effect of B. subtilis WT isolates and their NRPS deletion mutants against Fusarium and Botrytis, and further evaluation of two lipopeptides (surfactin and plipastatin) production, and prediction of secondary metabolite BGC by antiSMASH. The authors show the different antifungal capacity of B. subtilis correlates to the production of antifungal lipopeptides (surfactin and plipastatin) and their BGC content, and explain the genetic details of loss of the production of lipopeptide is based on nonsense mutation on the regulator process and lack of core biosynthesis genes. Besides the interspecies interaction, intraspecies interaction among these isolates is investigated and assumed to correlate to the BGC prediction result. Instead of single strain analysis, this manuscript shows that the similar B. subtilis can exhibit different antifungal capacity, based on the different production of potential antifungal lipopeptides and corresponding BGC content. This knowledge will foster an understanding of the ecological role of B. subtilis and transfers knowledge to the future biocontrol application.

Overall this research is well designed and the experiments are well performed and lead to the production of reliable data for sufficient interpretation and discussion. However, in order to make this research more suitable to publish on mSystems as high-quality research and match the broader readership, please see the following suggestions:

1) In the 'testing the antifungal potential of B. subtilis isolates' section, it is better to add the antifungal activity of P5-B2 and P8-B2 in figure 1A, since the gene cluster prediction from those two strains are discussed in figure 3. The difficulty of knockout (not naturally competent) can be commented into figure legend (Page 8, Line 164-166.
>The strains P5_B2 and P8_B2 were included in figure 2A and 2B (former figure 1) and non-transformable strains were mentioned additionally in figure legend (Fig. 2A).

2) Page 9, Line 173-Line 188: the result section regarding antifungal potential against B. cinerea is a bit confused and not easy to follow. Could be possible to group the isolates as (or something similar): sfp-dependent (most of the isolates) and partial sfp-dependent (64 and MB8_B7); under sfp-dependent, there are plipastatin-dependent (39, P8_B1, Pb_B3) and partial plipastatin-dependent (MB8_B1, MB11_B1, MB12_B1, MB12_B3, MB12_B4, P5_B1) and synergistic effect (38, 72, 75, 77). The outlier will be 73, MB9_B4 and MB9_B6, which represent weaker antifungal from WT isolates.
>The section was revised.

3) In the section 'synergism between plipastatin and surfactin...', the statement 'the deletion mutant screen in combination with chemical profiling suggested that the presence of either plipastatin or surfactin is sufficient for inhibiting the growth of B. cinerea', however it is not enough to explain the strain 64 and MB8_B7 and a bit contradiction with the following statement. Please rephrase this paragraph (Line 211-Line 220) to make it more concise.
>The section was revised.

4) As standards of surfactin and plipastatin are available (LCMS standard), the antifungal activity of reference compounds should be tested and added to figure 1A; the same experiment should be performed for combinations of surfactin and plipastatin! These experiments will be essential to support the findings of the knockout study to clarify the synergism between plipastatin and surfactin for B. cinerea inhibition.
>Unfortunately, we do not have the standard any more in our collection that was used to create the chemical profiles. Due to delivery problems of pure plipastatin (we waited for more than 2 months from Sigma), we could not perform antifungal activity tests of the standard compound plipastatin against B. cinerea. In view of covid19 restrictions, lack of pure compounds, and not to further delay the revision, we decided to remove any indication of synergism and refer that both compounds impact B. cinerea.

5) In Figure 2C, the targeted LCMS chromatograms of surfactin and plipastatin show multiple peaks, please clarify or mention whether they are isomers/analogs.
>See next comment.

6) In Figure 2C, it seems the multiple pattern of surfactin and plipastatin from B.subtilis isolates is different from the standards (when zoomed in). Please clarify the composition or explain the differences.
>Figure 1 and 2 were swapped. The multiple peaks show different surfactin and plipastatin analogs with different fatty acids substitutions. The figure legends of figures 1C (former 2C), S3 and S4 were revised.

7) Bacilysin is also reported as antifungal NRPS product and the BGC is widely spread in all B. subtilis isolates.

Please clarify or discuss whether the antifungal activity can from bacilysin or not, althrough bacilysin is not sfp-dependent NRP.
>The impact of bacilysin was implemented in the discussion.

8) In Figure 4A, please indicate the mutation of comA in MB9_B4 to show the reason of non-production of surfactin in the Figure 4A (Page 12, Lin 263). It is better to present in the main text instead of SI.
>We have no direct proof that ComA protein product is reduced, therefore we prefer to leave this information in the supporting material. Thus, this mutation in comA could be one possibility but is not a proof of our hypothesis.
We will rely on the decision of the Editor whether it is a must to include this adjunct information in the main results. Also, the text clearly indicates the changes, the alignment does not provide too much essential information, thus better be placed in the supplementary file.

9) The section of 'intraspecies interaction' is not well interpreted and even no discussion about the results is included. The only prediction of potential BGC is not sufficient to explain the interaction assay (Figure 5).
>A discussion of the intraspecies interaction section was included.

10) Page 11, Line 224: please comment the 'in addition to one *B. licheniformis* strain used as an outlier' is P8_B2).
>The section was revised.

11) Page 11, Line 226: please clarify 'all predicted BGCs' or 'all predicted peptidic BGCs'. It is better to mention whether some other gene clusters are present or not, in order to discuss the overall contribution of secondary metabolites (or their BGCs).
>Fig. 3 gives an overview about all predicted BGCs, which show similarity to known gene clusters. However, we have mentioned the unknown predicted BGCs of the B. subtilis strains at the end of this chapter.

12) Page 5, line 99, please clarify or give full name of sfp for the first time.
>The section was revised and Sfp was explained when mentioned the first time.

13) Page 12, line 263, please clarify or give full name of comA for the first time.
>The introduction of ComA was revised.

Reviewer #2 (Comments for the Author):

This work provides interesting results showing that different Bacillus strains of the same subtilis species are not equal regarding their potential to produce cyclic lipopeptides as important bioactive secondary metabolites. It is quite unexpected considering the recent genomic analyses correlated with phylogeny from which it can be deduced that B. subtilis is invariabily a producer of surfactin and fengycin/plipastatin. Not easy to explain but it suggests an underestimated effect of environmental pressure in shaping the genetic basis for lipopeptide production...
The work has been seemingly well conceived and the manuscript is generally well written but I have some remarks and concerns listed below.

L70-74: I would agree with those general statements but the authors should use more recent reviews! I'm not sure that Straight et al. showed any role as signal for lipopeptides in that study...
>References were rearranged.

L78-80: Taxonomy has been adapted in the recent years and additional species (velezensis, atrophaeus) have been included in the B. subtilis complex. Again, the authors should refer to more recent papers at that place such as Harwood et al that is actually used later in the Introduction (L97)!!
>Novel strains were included, and a recent publication was used (Caulier et al., 2019)

L110-112: an additional example of un-appropriate use of reference works with a review and biophysic studies that are not really supporting antibacterial activity at least via leakage. The authors should use research papers unambiguously describing antimicrobial activity of surfactin based on cytolytic activity when biologically relevant concentrations (low micromolar) are tested.
>The references were revised and additional research studies addressing the cytolytic activity towards bacteria and additional functions were included.

L119: does the distinction between plipastatin and fengycin really matter? In other words, does the very small change in structure between the two compounds (isomery of one amino acid in the middle of a 10 residue peptide) significantly impact antimicrobial potential or bioactivity in general?
>The small difference in only one peptide moiety between plipastatin and fengycin does not affect their bioactivity. In literature both names are used, but a consistent use of the correct name is desirable. Furthermore, it has been shown, that the gene cluster of plipastatin is primarily found in B. subtilis, and the fengycin gene cluster in B. amyloliquefaciens/B. velezensis. We now included citation to recent manuscript that examines gene clusters in 310 isolates from the B. subtilis group, clearly demonstrating that the gene clusters are distinct.

L124: Add "direct" before antimicrobial properties since it's a siderophore...
>The sentence was revised.

L128: I don't understand the word "systematically" here
>"systematically" was omitted and the sentences was revised.

L133: why testing two different Fusarium and not another very different fungus together with Botrytis?
>We tested the two Fusarium spp. because of their availability in our strain collection. Even though the screening results were similar, these plant pathogens have different host plants and results are interesting for future biocontrol applications or improvement.

End of Introduction: there is not reference to the last part of the work on interactions between bacilli! Maybe the authors do not consider it as crucial in the context of this work... and I would agree!
>A reference was included at the end of the introduction.

L173-188: The authors should refer once more to the figures in this long piece of text...
>Section was revised and an additional reference to the figure was added.

Paragraph L195-209: Is it a similar growth for all strains on PDA plates? It's not easy to accurately determine but at least upon visual assessment? Sampling was performed in the colony? In the medium around? The way and place plugs are performed in the gelified medium may be crucial... Plipastatin peak is very low in Fig 1C... Is it really possible to discriminate/classify/compare the different strains for lipopeptide production (or better production rate) in these conditions?
>The colony size was monitored by eye and a variability in colony size among the WTs was observable. In general, surfactin mutants displayed a reduced colony size due to lack of surfactin and therefore reduced expansion ability of colonies. However, we could not reveal that colony size affected the inhibition potential.

The amount of plipastatin was compared among strains, but also the presence or absence of MS peaks was also used to claim that no plipastatin was produced in selected isolates. Nevertheless, we did not quantify the exact amount of plipastatin in this study, rather present a presence or absence of plipastatin production.

L219: what do you mean by "intermediates" ? Other products from incomplete synthesis that are antifungal? It would be surprising for that kind of compound when we know the importance of a "mature" structure with entire and cyclized peptide linked to the fatty acid for bioactivity...
>"Intermediates" was omitted and the section was revised.

L233: what is the reference?
>The reference strains are for B. subtilis various B. subtilis strains depending on which reference cluster is used by antiSMASH. These strains are defined in antiSMASH database.

L325-326: Again, the references used do not really support the statements: a review, a biophysic paper (... on fengycin, does it work the same as plipastatin?) and the quite old paper which described the phenomenon (phospholipase inhibition) but never supported or confirmed by other studies later on...
>We thank the reviewer for this important suggestion. We decided to remove the sentence to avoid citing a phenomenon that never been supported by later studies.

L333: "potential intermediates": knowing the gene that was disrupted and according to the co-linearity rule, those possible intermediate products may be predicted no? What is the predicted structure? size? still cyclic?
>We have not investigated the potential intermediate compounds in more detail in this project. This is outside of the scope of the current manuscript.

L335-359: not useful!
>In this section, we describe that domestication will affect SM production as in case for the laboratory strain 168. Furthermore, we find it important to explain why surfactin production could be reduced (comA) and discuss the possibility that it is affected by the point mutation in comA.

L379: "ambiguous": what does it mean, no real roles? contradictory with previous statements placed several times in the manuscript.
>The word "ambiguous" was omitted and sentence was rephrased.

What about the presence of BGCs (presumably silent under lab conditions) coding for undescribed NRPS products with possible antifungal activity? No prediction with Antismash?
>There are no further predictions for NRPS gene clusters by antiSMASH. While B. subtilis is well characterized, gene clusters have been examined that could possibly code for NRPS.

Reviewer #3 (Comments for the Author):

In their work the authors have isolated 22 B. subtilis strains from various locations in Germany and Denmark for investigating their inhibition potential against the plant pathogenic fungi Fusarium oxysporum and graminearum as well as Botrytis cinerea. The antifungal potential of these organisms mainly depends on the action of their lipopeptide products plipastatin and surfactin. For prediction of the biocontrol effects of the B. subtilis isolates mass spectrometric product detection, mutational analysis with sfp as well as srf and pps defect mutants and genome analysis of the biosynthetic gene clusters were combined. It was shown that plipastatin efficiently inhibited the Fusarium strains, while both plipastatin and surfactin contribute to full inactivation of Botrytis cinerea.
13 of the B. subtilis isolates were selected for genome sequencing. Their potential for the production of bioactive compounds was derived from AntiSMASH 5.0 analysis specifying the biosynthetic gene clusters detected in the genomes of these organisms, but the products that are ultimately formed, can only be determined by mass spectrometric and chemical analysis. In this context the authors should comment which of the compounds listed in Fig. 3, as for example, subtilosin A, subtilomycin, sublancin, subtilin and butirosin are really expressed. Evaluation of the pps gene clusters in the genomes of plipastatin nonproducer strains revealed the reduction or complete loss of pps genes. The authors argue that in the case of BGC mutants potential intermediates might be produced that affect fungal growth. Did the authors attempt to detect, purify and characterize such intermediates?
>The purification of potential intermediate requires further work and is ongoing in our laboratories.

The following figures and their legends should be modified:

Fig. 1B Please, specify SM production in the legend.
>Fig.1 and Fig. 2 were exchanged. In Fig. 2,*”SM production” was changed to “NRP”* and the figure legend was revised.

Figs. 2C; S3 and S4C: the numbers and designation at the coordinates are too small. In particular, the titles at the abscissa are difficult to read.
>Fig.1 and Fig. 2 were exchanged. The axis labelling of chromatograms in figures 1C, S3 and S4C was increased as much as possible. Additionally, the chromatograms in figure S3 were rearranged.

Fig. S2 Please, specify in the legend the positions of the single NRP mutant in the middle part of this figure.
>Position of strains was mentioned in figure legend.

Fig. S3 Delete iturins in the legend, because they are not products of B. subtilis.
>Figure legend was revised.

Fig. 4 The deficiency of surfactin formation by MB9_B4 (-) should be explained in the legend.
>Figure legend was revised.

Fig. 5A is difficult to understand and should be better explained in the legend.
>The figure legend of 5A was revised.

Fig. S5 Is MB9_B6 in the legend correct? For my opinion it should be replaced by MB9_B4 !
>Yes, MB9_B6 is correct. This strain has a point-nonsense mutation in the ppsB gene, while MB9_B4 is producing plipastatin.

Amendments in the text:

Page 8, line 146: A library of 22 B. subtilis isolates....
>The sentence was revised.

Page 10, line 200 Where is Fig. 1C? It does not exist.
>Figure 1 and 2 were swapped, Fig. 1C now exists.

Page 10, line 201 Fig. 2B is not correct? Surfactin and plipastatin were detected in Fig. 2C!
>Figure 1 and 2 were swapped. Fig.2B gives an overview about the surfactin and plipastatin production of each wild type strain.

Page 16, line 318 microorganisms
>Macroorganisms are correct, but we complemented it with microorganisms.

Page 17, line 350 extent
Page 17, line 343 better: model strains have rapidly lost their ability.....

>The sentences above were revised.

Page 23, line 483 intermicrobial
Page 24, line 508 peptidyl carrier protein
>The typos in the references were revised.

January 27, 2021

Prof. Ákos T Kovács
Technical University of Denmark
Department of Biotechnology and Biomedicine
Søltofts Plads 221
Kgs Lyngby 2800
Denmark

Re: mSystems00770-20R1 (Genomic and chemical diversity of *Bacillus subtilis* secondary metabolites against plant pathogenic fungi)

Dear Prof. Ákos T Kovács:

Your manuscript has been accepted, and I am forwarding it to the ASM Journals Department for publication. For your reference, ASM Journals' address is given below. Before it can be scheduled for publication, your manuscript will be checked by the mSystems senior production editor, Ellie Ghatineh, to make sure that all elements meet the technical requirements for publication. She will contact you if anything needs to be revised before copyediting and production can begin. Otherwise, you will be notified when your proofs are ready to be viewed.

As an open-access publication, mSystems receives no financial support from paid subscriptions and depends on authors' prompt payment of publication fees as soon as their articles are accepted. You will be contacted separately about payment when the proofs are issued; please follow the instructions in that e-mail. Arrangements for payment must be made before your article is published. For a complete list of **Publication Fees**, including supplemental material costs, please visit our website.

Corresponding authors may join or renew ASM membership to obtain discounts on publication fees. Need to upgrade your membership level? Please contact Customer Service at Service@asmusa.org.

For mSystems research articles, you are welcome to submit a short author video for your recently accepted paper. Videos are normally 1 minute long and are a great opportunity for junior authors to get greater exposure. Importantly, this video will not hold up the publication of your paper, and you can submit it at any time.

Details of the video are:

· Minimum resolution of 1280 x 720
· .mov or .mp4. video format
· Provide video in the highest quality possible, but do not exceed 1080p
· Provide a still/profile picture that is 640 (w) x 720 (h) max

We recognize that the video files can become quite large, and so to avoid quality loss ASM

suggests sending the video file via https://www.wetransfer.com/. When you have a final version of the video and the still ready to share, please send it to Ellie Ghatineh at eghatineh@asmusa.org.

Thank you for submitting your paper to mSystems.

Sincerely,

Matthew Traxler
Editor, mSystems

Journals Department
American Society for Microbiology
1752 N St., NW
Washington, DC 20036
E-mail: peerreview@asmusa.org
Phone: 1-202-942-9338

Supplemental Table S1: Accept
Supplemental Figure 3: Accept
Supplemental Figure 5: Accept
Supplemental Table S2: Accept
Supplemental Figure 4: Accept
Supplemental Figure 1: Accept
Supplemental Figure 2: Accept
